# Can adult weights be used to value child health states? Testing the influence of perspective in valuing EQ-5D-Y

**DOI:** 10.1007/s11136-015-0971-1

**Published:** 2015-04-19

**Authors:** Paul Kind, Kristina Klose, Narcis Gusi, Pedro R. Olivares, Wolfgang Greiner

**Affiliations:** Academic Unit for Health Economics, Institute for Health Sciences, University of Leed, Leeds, UK; Department of Health Economics and Health Care Management, Bielefeld University, Bielefeld, Germany; Exercise, Quality of Life and Health Economics, Faculty of Sport Sciences, Universidad de Extremadura, Cáceres, Spain

**Keywords:** EQ-5D, EQ-5D-Y, Valuation of health, Children

## Abstract

**Purpose:**

To test whether or not adults assign the same values to hypothetical health states that describe health in adults as when those same descriptions refer to the health of a child.

**Methods:**

A two-part self-completion questionnaire was designed in which respondents were asked firstly to rate a fixed set of EQ-5D-Y health states on a 0–100 visual analogue scale as if they themselves were in these states. Two versions of the questionnaire were produced each with a different second part. One version instructed respondents to value the same states but to imagine them describing another adult. The second version required respondents to value these states as if they applied to a 10-year-old child. Questionnaires were distributed to adults recruited in three countries (Germany, Spain and England) using convenience sampling methods.

**Results:**

A total of 1085 questionnaires were completed. Despite some significant differences in the characteristics of the achieved samples in the three countries involved, the rank order of health states was largely consistent across each adult/child reference perspective. In all countries, the mean values were lower when health states described children rather than adults. Significant differences were found for 16/24 states when values for those states applied to adult respondent themselves were compared with the values for those states applied to a 10-year-old child. A near-uniform pattern was found across all three countries in which health state values for children were found to be lower than for adults.

**Conclusions:**

Values for health states when ascribed to adults are higher than when those same states are associated with children. Were EQ-5D-3L values for adults applied to EQ-5D-Y health states, then this would effectively lead to an misrepresentation of the value assigned to a health status in children.

## Background

The measurement of health benefit is arguably the central issue in any evaluation of healthcare interventions. The selection of outcome measures in such situations is largely determined by the type of evaluation being conducted and the decisions likely to be informed thereby. For the evaluation of adult interventions, there is a small, well-defined set of generic index measures that are in widespread use, foremost amongst these being EQ-5D [1]. However, the situation is far less clear when it comes to the measurement of health benefits in children where the development of appropriate metrics has significantly lagged behind the corresponding effort directed towards developing adult measures. This differential can in part be attributed to wider and more deep-rooted issues that have obstructed progress in this specialist research field. The complexity of developing generic measures for use with children and young people has parallels in long-standing issues that have attended the development and use of health status measures in other groups such as the elderly and those with communication problems or high levels of dependency.

The rate and nature of child development, especially in the very young, is a major confounding factor in identifying and measuring changes in health status—especially where health status is predominantly defined in terms of independent function. How much of any registered change in health status might result from external interventions and how much is attributable to intrinsic developmental change, some of which may be nonlinear and in some situations even regressive? Does health status in children have conceptual or ultimately empirical correspondence with health status as conceived and measured in adults? When capturing descriptive information on child health status then whose assessment should be used—the child him/herself, a parent or carer? As for the determination of the value or worth to be associated with a child’s health, this question alone has the capacity to provoke debate across broad swathes of any society.

The economic evaluation of health and social care interventions in childhood is associated with a broad range of methodological issues the majority of which lack the formal guidance that applies to technology assessment in adults. This paucity has been noted by several observers [2–4] who draw attention to the questionable methods currently employed in determining utility weights in economic evaluation. Much of this variable practice seems to be fuelled by the pressing need for quality-adjustment estimates for use in generating evidence of cost-effectiveness on the one hand, and the near-universal absence of such estimates reported in the scientific literature on the other. The use of social preference weights for use in valuing health in children but elicited using descriptions of health states in adults introduces untested assumptions that are not consistent with acceptable scientific standards. The case for collecting utilities directly from children can be readily put aside in those decision-making settings where a societal perspective is adopted and where social preferences weights are used rather that those of the patient or other beneficiary. However, it is less clear that any single method has been established by which to elicit utility weights for health in children nor is it completely evident as to how such a choice should be made between competing techniques such as standard gamble (SG), time trade-off (TTO) and discrete choice (DC) methods [5, 6]. A more problematic issue relates to the extent to which “health” in children is regarded as being of intrinsically greater value than in adults. One study in which stated preference methods were applied to standardised health state descriptions linked to hypothetical individuals of different ages found that “that the public places a greater value on preventing outcomes in children, compared to adults” [7]. This phenomenon generalises to choices made between competing health programmes. A survey of US adults found that preferences for health gains for children that go well beyond differentials that can be explained by relative life expectancy [8].

EQ-5D-Y [9] is a generic measure of health status in children and young people with a design architecture analogous to that of the original three-level version of EQ-5D (EQ-5D-3L) used with adults. Both systems define a total of 243 health states based on five dimensions each with three problem levels (none, some, extreme); however, there are some subtle but important differences between the two classification systems. EQ-5D-Y was developed following initial efforts to modify the language and content of the adult EQ-5D and render it usable by children of school age [10]. This work led ultimately to the revision of some dimension and response labels. The practical feasibility and validity of EQ-5D-Y were subsequently demonstrated in several reported studies [11–13].

For the purposes of cost-utility analysis based on quality-adjusted life years (QALYs), the original adult version of EQ-5D can be represented as a single utility-weighted index using social preferences derived from the general population. Scoring systems based on social preferences for EQ-5D health states have been elicited in a number of countries. The evolution of a second-generation EQ-5D classification with five levels of response (EQ-5D-5L) [14] has stimulated the development of innovative valuation methodologies capable of being used with a more complex descriptive system. The EQ-5D-Y health state classification by contrast currently lacks any corresponding scoring system capable of representing health state values as a single index. The design of any valuation study involves the consideration of significant technical issues—for example, the number and composition of health states to be included and the need to take account of the experimental burden placed on participants in completing the valuation tasks with which they are confronted. A further complication in the design of any study that examines the valuation of health states in children is that of the perspective of the respondent. The valuation of EQ-5D health states in adults has predominantly required participants to assess descriptive profiles that were attached to themselves or at any rate, a person like them. Values for these “hypothetical” states can and do vary when data are analysed on the basis of respondent characteristics such as age or social class. Little appears to be known about the effect of perspective on the issue of valuation, by which is meant the position of the individual in relation to the hypothetical health state that is imagined. EQ-5D health state values are considered applicable to adults of all ages (in theory aged from 16 to 100 years). For these purposes, it is assumed that by requesting participants to imagine health states as though they described “a person like you”, any age specificity in the health state description is avoided. In the case of EQ-5D-Y health states, if this traditional approach was taken, then respondents would be asked to adopt a more complex perspective in which they would be asked firstly to imagine being a child and then secondly to superimpose a given EQ-5D-Y health state on that image.

The EQ-5D-Y health state classification currently lacks a scoring system based on social preference weights. Such a value system is needed if EQ-5D-Y is to be used for QALY calculations in cost-utility analysis, but there are significant costs associated with the development of any value set as well as particular conceptual and methodological issues linked to the valuation of health in children and young people. An interim solution therefore might be to apply adult EQ-5D value sets to the EQ-5D-Y classification, but evidence to support the legitimacy of such a move is lacking. The principal objective of this present study was to test the effect of perspective in valuing EQ-5D-Y health states and to examine two questions in particular—do the values for hypothetical health states change when adults are asked to imagine another adult in that state as opposed to themselves; does the value for a health state change if a child is described as being in that state rather than the adult respondent.

The core issue is whether or not in adult respondents, valuations for hypothetical health states are altered when those states describe a child/young person rather than the respondent themselves. If there is no difference in elicited values, then other things being equal, it might be possible to apply social values derived from an adult population in weighting health status in children. The main point of this study is less about the magnitude of any differences in values, but rather whether values for adult health states differ at all from the values for the same health states when associated with a child. If values do change, then a secondary issue concerns the magnitude of any observed differences and whether they can be accounted for by any observed respondent characteristics. These issues impact on the more general question as to whether valuation tasks should require participants to value health in themselves or in others. More narrowly, the study provided an empirical basis from which to consider an emerging question provoked by the absence of an existing value set for converting EQ-5D-Y data into a single utility-weighted index, namely could EQ-5D-3L weights be applied as an interim expedient.

## Subjects

The survey was fielded concurrently in three countries (Germany, Spain and England) to avoid reliance on results from a single source. In each case, a convenience sampling strategy was adopted. Since the focus of the study was the investigation of a single methodological issue and was not intended as means of generating a set of social preference weights for EQ-5D, there was no necessity to ensure that respondents should constitute a nationally representative sample. Furthermore, had the study been designed with the intention of generating weights for EQ-5D, then the number of states selected for inclusion in the protocol would have needed to be correspondingly higher.

## Materials

A self-completion multi-section questionnaire was designed (a copy of the English version is appended^1^). Respondents were asked to describe and rate their own health state using EQ-5D-3L before being presented with a set of eight EQ-5D-Y health states (three mild, two moderate and three severe health states) selected across its full severity range and printed on a single page in two groups of four, arrayed either side of a vertical 20-cm VAS rating. The endpoints of the scale were labelled “best imaginable” and “worst imaginable”. Respondents were instructed to write the value for each state in a marked box adjacent to the health state description. Instructions for completing this page direct the respondent to consider each state as if applying to themselves (SELF) and that this state would last for 1 year. No further information was given to the respondent regarding future health status beyond that time horizon. The same set of states was presented again in the following section, but here the respondent was instructed to interpret the descriptions as applying to someone else. In half of the questionnaires (version A), this other person was stated as being another adult (ADULT). In the remaining half (version B), respondents were asked to think of the states as applying to a 10-year-old child (CHILD). A common set of background questions was included in both versions. The questionnaire concluded with a series of open-ended questions inviting respondents to comment on the completeness of the EQ-5D-Y descriptive system, the extent to which adult and child health are comparable and whether or not priority should be given to improving health status of children rather than adults. It should be noted that a printing error resulted in one health state appearing in the English version that did not correspond to the standard set adopted in Germany and Spain.

## Data

Data from the three survey sources were merged into a single data set which was analysed using IBM Statistics SPSS 22. Free-text responses collected in this study will be reported separately.

## Results

A total of 1085 questionnaires were received in response to the three surveys, and the general characteristics of the respondents are shown in Table 1. There are some obvious differences—respondents in Germany tend to be somewhat older than those in Spain or England; those in Spain and the England were more likely to be women than those in Germany. In terms of age distribution, there is a markedly younger sample in Spain where 70 % of respondents are aged under 40 years compared with the 30 % seen in the German sample.

**Table 1:** Characteristics of study samples

	Germany	Spain	England
Number of respondents (% female)	407 (55.4)	441 (67.6)	237 (60.1)
Age of respondent (median)	49 years	32 years	37 years
0–19	2 (0.5 %)	0 (0 %)	9 (3.8 %)
20–29	68 (16.9 %)	189 (42.9 %)	62 (26.5 %)
30–39	55 (13.6 %)	122 (27.7 %)	65 (27.8 %)
40–49	78 (19.4 %)	106 (24.0 %)	30 (12.8 %)
50–59	91 (22.6 %)	19 (4.3 %)	40 (17.1 %)
60–69	47 (11.7 %)	2 (0.5 %)	20 (8.5 %)
70+	62 (15.3 %)	3 (0.7 %)	8 (3.4 %)
Marital status			
Married/living with partner	268 (65.8 %)	206 (46.7 %)	167 (71 %)
Divorced/separated	36 (8.8 %)	29 (6.6 %)	20 (9 %)
Single	73 (17.9 %)	201 (45.6 %)	32 (14 %)
Widowed	28 (6.9 %)	5 (1.1 %)	11 (5 %)
Level of education			
Basic schooling	44 (10.8 %)	46 (10.4 %)	73 (30.8 %)
Intermediate	204 (50.1 %)	201 (45.6 %)	69 (29.1 %)
Higher/professional	148 (36.4 %)	194 (44.0 %)	82 (34.6 %)
Economic status			
Employed/self-employed	244 (60.0 %)	235 (53.3 %)	139 (59 %)
Retired	90 (22.1 %)	6 (1.4 %)	61 (26 %)
Housework	22 (5.4 %)	37 (8.4 %)	15 (6 %)
Student	35 (8.6 %)	137 (31.1 %)	2 (1 %)
Unemployed	6 (1.5 %)	26 (5.9 %)	10 (4 %)
Other	6 (1.5 %)	0 (0 %)	3 (1 %)
Parenting experience	270 (66.0 %)	265 (60.0 %)	193 (82.8 %)
Work with children	87 (21.4 %)	288 (65.3 %)	39 (16.5 %)

The proportion of English respondents married or living with a partner is much higher than in Spain where single respondents predominate. Almost one-third English respondents have only received basic schooling—some three times the proportion seen in the Spanish and Germany studies.

Economic status across the three studies is broadly similar in terms of the proportions who are currently in work. The minute level of retired respondents in the Spanish sample is entirely consistent with the skewed age distribution reflecting the high proportion of students. The different age distributions seen in the three samples most probably accounts for the significantly different rates of parenting experience with 83 % of English respondents reporting personal experience as parents compared with 66 % for Germany and 60 % for Spain. Similarly, the distribution of respondents with current experience of working with children varies across the three samples Spain recording 65 %, Germany 21 % and England 16 %.

The health status of the three samples seems much higher amongst English and Spanish respondents with more than 50 % reporting their health to be excellent/very good in both instances, compared to the 36 % of German respondents. A much higher proportion of English respondents than in Spain or Germany indicate that their health status is only fair/poor.

Levels of reported problem for each of the EQ-5D dimensions are also presented in Table 2, and it can be seen that virtually all respondents in Spain report no problems with mobility, a finding in line with age distribution in that sample. In fact, this is a pattern that extends across all dimensions and it is therefore unsurprising to see that the EQ-5D_VAS_ self-rated health status is higher in this sample compared with the English and German studies.

**Table 2:** Self-reported health status in study samples

	Germany	Spain	England
Self-rated health			
Excellent	26 (6.4 %)	80 (18.1 %)	29 (12 %)
Very good	122 (30.0 %)	167 (37.9 %)	89 (38 %)
Good	212 (52.1 %)	37 (8.4 %)	73 (31 %)
Fair	34 (8.4 %)	15 (3.4 %)	31 (13 %)
Poor	3 (0.7 %)	1 (0.2 %)	8 (3 %)
Missing	10 (2.5 %)	0 (0 %)	4 (2 %)
Problem level on EQ-5D-3L			
Mobility			
Level 1	351 (86.5 %)	431 (97. 1 %)	195 (82.3 %)
Level 2	54 (13.3 %)	10 (2. 3 %)	38 (16.0 %)
Level 3	1 (0.2 %)	0 (0 %)	0 (0 %)
Self-care			
Level 1	391 (96.5 %)	437 (99. 1 %)	220 (92.8 %)
Level 2	12 (3.0 %)	3 (0. 7 %)	8 (3.4 %)
Level 3	2 (0.5 %)	1 (0. 2 %)	4 (1.7 %)
Usual activity			
Level 1	365 (89.9 %)	422 (95. 7 %)	192 (81.0 %)
Level 2	38 (9.4 %)	19 (4. 3 %)	34 (14.7 %)
Level 3	3 (0.7 %)	0 (0 %)	6 (2.6 %)
Pain/discomfort			
Level 1	223 (55.2 %)	351 (79. 6 %)	147 (63.4 %)
Level 2	172 (42.6 %)	88 (20. 0 %)	81 (34.9 %)
Level 3	9 (2.2 %)	2 (0. 5 %)	4 (1.7 %)
Anxiety/depression			
Level 1	304 (74.9 %)	374 (84. 8 %)	171 (73.4 %)
Level 2	96 (23.6 %)	64 (14. 5 %)	56 (24.0 %)
Level 3	6 (1.5 %)	3 (0. 7 %)	6 (2.6 %)
Any missing	0.2–0.7 %	0–0.7 %	1.7–2.4 %
EQ-5DVAS			
Mean	79.5	85.5	79.3
Median	80	90	80

Face validity checks on the valuation data during encoding triggered a more formal scrutiny of data quality. Respondents who assigned an identical value to all health states were presumed to have failed to comprehend the task required of them. For inclusion in the analysis of the aggregated valuations, data respondents were required to have no more than four missing values and to have assigned at least four different values to health states. Data attrition accounted for around a 20 % loss of respondents.

The mean values for health states in each of the three study samples are given in Table 3. The rank order of states within each sample remains remarkably similar regardless of which perspective is specified. The general pattern of the German data is clear with the values for all eight health states being higher when applied to another adult (ADULT) than when the health state applied to the respondent themselves (SELF)—a pattern that is broadly similar to the mean values seen for 7/8 states in the survey from Spain. In the English data, however, there is a wholly different pattern with an increased value seen for only 2/8 (SELF) states. None of the recorded respondent characteristics accounted for this differential pattern. Nevertheless, in all countries, there is a near uniform lowering of mean values when health states are associated with a child (CHILD) rather than with the respondent themselves or with another adult.

**Table 3:** Mean values for EQ-5D-Y health states

EQ-5D-Y state	Germany	Spain	England
SELF			
11121	85.639	88.895	87.394
33232	24.652	40.263	29.964
32233	19.798	33.491	25.057
21312	43.924	44.541	42.580
12232	38.457	45.781	
12121			62.121
21122	55.734	53.187	56.847
11122	69.279	62.235	70.115
33223	21.050	22.610	24.143
ADULT			
11121	86.055	88.276	82.185
33232	27.000	42.195	30.660
32233	21.017	34.681	24.726
21312	45.194	45.619	39.679
12232	38.084	47.686	
12121			64.252
21122	57.391	55.838	55.822
11122	70.911	63.948	66.290
33223	21.466	23.833	20.972
CHILD			
11121	81.202	86.947	79.812
33232	20.694	39.513	22.855
32233	15.417	32.430	20.012
21312	37.076	43.311	34.560
12232	32.414	42.925	
12121			62.381
21122	48.955	48.618	54.085
11122	63.372	56.882	60.318
33223	16.064	20.452	20.536

**Fig. 1:**
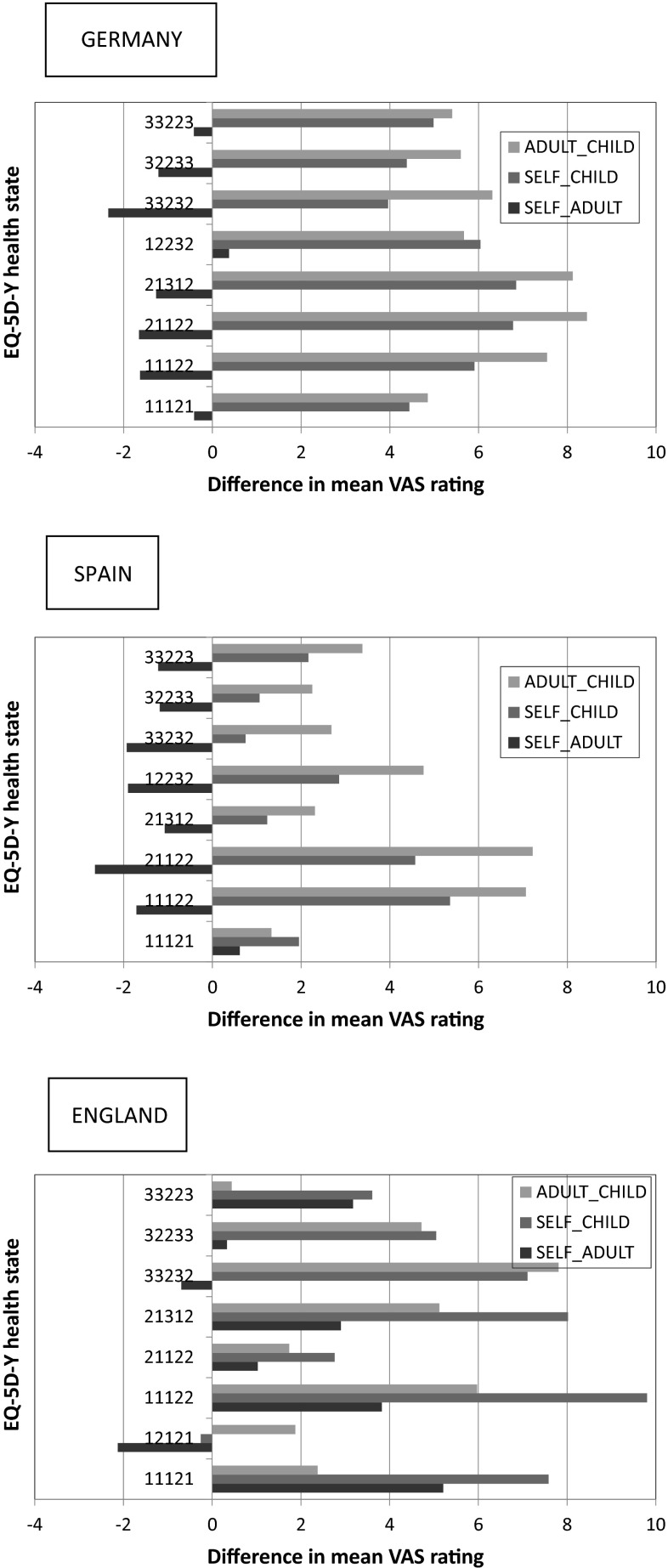
Differences in value for EQ-5D-Y health states according to respondent perspective

Within-respondent differences in values for SELF-ADULT and SELF-CHILD were analysed using paired t-tests, separately for each country. Table 4 shows the mean differences in value for EQ-5D-Y health states, and these are presented graphically in Fig. 1. Statistically significant differences are seen for 4/24 states across the whole study sample when SELF values are compared with ADULT values (Germany 1, Spain 1, England 2). When SELF values are compared with CHILD values, 16/24 states are statistically different (Germany 8, Spain 3, England 5).

**Table 4:** Differences in mean values for EQ-5D-Y health states

SELF-ADULT	Germany	Spain	England	SELF-CHILD	Germany	Spain	England
11121	−0.133	1.667	5.708***	11121	4.727***	1.022	7.962***
33232	−2.568**	−2.067	−1.294	33232	3.962***	0.873	5.949***
32233	−1.114	0.362	−1.020	32233	4.613***	−0.368	4.130*
21312	−0.139	−1.938	2.126	21312	5.269***	2.022	6.987***
12232	−1.045	−2.324	–	12232	6.731***	3.241*	
21122	−1.274	−2.824*	2.058	21122	6.948***	4.728***	2.922
11122	−1.842	−0.757	4.596*	11122	5.523***	4.474***	8.513***
33223	−1.074	−1.281	1.861	33223	5.632***	2.211	3.667
12121			−0.433	12121			−1.526

ns signifies *p* > 0.05

* *p* < .05; ** *p* < .01; *** *p* < .005

The distribution of absolute values of all the means reported in Table 4 reveals that a value of 5.0 distinguishes the upper 20 % of differences. A total of ten pairs of states record differences that exceed this value of which one is recorded for SELF-ADULT values, and the remaining nine are for SELF-CHILD values. This latter pattern is further compounded by the observation that of these, five differences are seen in the German SELF-CHILD values and four in the equivalent English data; none of the differences observed in the Spanish SELF-CHILD data exceed this selected cut point.

**Fig. 2:**
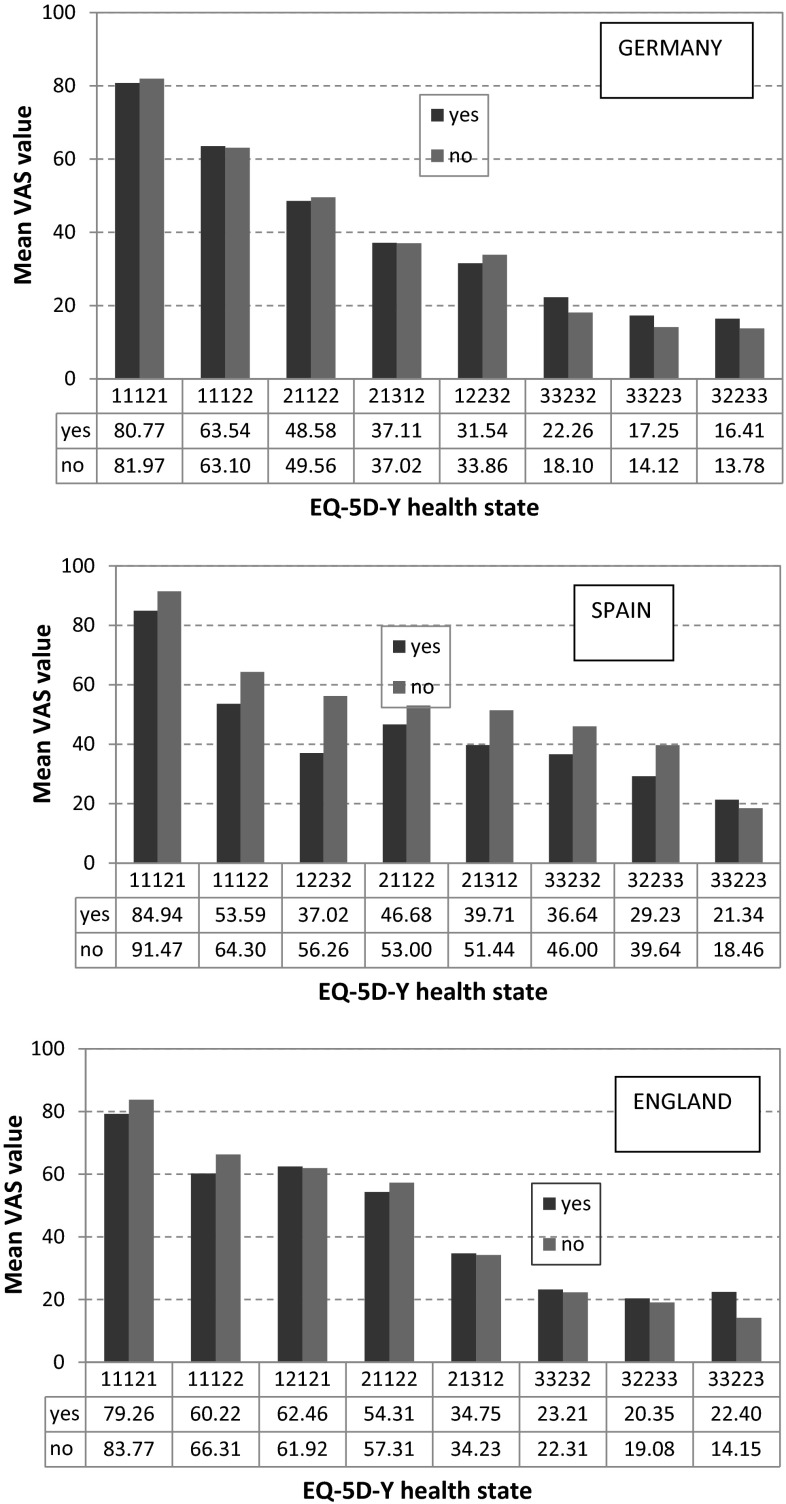
Parenting experience and health state valuation of child health states

As this study is concerned primarily with perspective, values for those respondents with/without parenting experience were computed separately for each country and the results are shown graphically in Fig. 2. In the German data, it appears that parenting experience is associated with small differences ranging from −4 to +2 points on the VAS, with 4/8 states being valued more highly by respondents with parenting experience than those without. In the Spanish data, there is a more uniform pattern of differences. Respondents with parenting experience assigning lower values than non-parents for 7/8 states; these differences largely in the range −1 to +15 points. In the English data, the pattern is similar to that seen in the German sample; however, differences are somewhat larger ranging from −7 to +13. The association of parenting experience was further examined by computing the differences in SELF and CHILD values for each state and applying a series of one-way analysis of variance tests using each of the respondent characteristics as the grouping factor. No evidence of any systematic association was found. These differences were also analysed using regression analysis in which respondent characteristics were coded as independent dummy variables, with similar results being obtained.

## Discussion

This study involved a common questionnaire-based methodology in which respondents initially valued a set of health states as they related to themselves. Half the survey then valued those same health states as though they related to another adult; half the survey valued the same health states as though they related to a 10-year-old child. The effect of order of presentation was not controlled for in the design of this study, and whilst it is conceivable that such order effects might be encountered, it is worth noting that the order of presentation was fixed for both versions of the questionnaire so that the influence of such a factor could reasonably be held to be similar in both cases. In ideal circumstances, it would have been useful to have extended the questionnaire variants to reverse the order in which ADULT and CHILD states were presented.

Participants in all countries were recruited using convenience sampling methods leading to achieved samples that differ to some extent in terms of a range of characteristics which might account for some of the observed variation in respondents’ values. Opportunistic sampling methods introduce the potential for a skewed sample with consequential implications for statistical analysis. Given the essentially self-selecting approach to recruitment, a degree of caution is required in interpreting results—not least of these reasons being the possibility that respondents (whether parents or not) were likely to have a stronger interest in children than non-respondents. It would be equally unjustified to suggest that data are representative of any single country.

However, it should be borne in mind that the focus of attention in this study was primarily the identification of any within-respondent shift in values. The finding that values for hypothetical health states attached to adult respondents themselves or to other adults are not statistically significantly different is perhaps unsurprising; however, the pattern of differences suggests that English respondents are at odds with their German and Spanish counterparts. Whether this represents stoicism or altruism is difficult to establish given the limitations of the survey data. It is nevertheless an interesting result that might be worth taking into account for future research especially in framing valuation studies for adult hypothetical states. Despite the evident heterogeneity of the survey data, one single unambiguous finding stands out across the three study samples, namely that the value for a health state when it is applied to a child is lower than that which is elicited when that same state is used to describe an adult’s health. The near uniformity of this finding is striking and suggests that the use of adult EQ-5D-3L weights to score EQ-5D-Y health states risks misrepresenting the value attached to health status in children. However, since these metrics are most often used in economic evaluation to value change in health benefit over time, then any systematic differences might conceivably be washed out when they are used to compute marginal QALYs in an incremental cost-effectiveness ratio. Of course, this assumes that the results seen in this study, which are based on VAS ratings, can be generalised to other preference elicitation methods such as TTO.

The presence of statistically significant value differences when adults assess health states ascribed to children might be dismissed as an interesting finding, but one that lacks any real relevance since for some states the value differences might be described as small and by inference of no real importance. This takes us into uncharted territory—at least as far as VAS ratings are concerned—in which the concept of a minimally important difference (MID) acts as a threshold that operationalises a judgement as to whether or not a change score has serious or trivial implications. Guidance as to the relevant MID for VAS ratings, especially those used to value health-related quality of life, has been observed to be remarkably thin [15]; not least of the related issues here is the sheer multiplicity of different formats that are loosely categorised as being “visual analogue scales”. It appears unlikely that a single decision rule can be applied to all VAS ratings used to value health states since “importance” is properly established in terms of the specific application in which such data occur. Furthermore, the absence of larger value differences in the Spanish SELF-CHILD data seen in this study suggests that these could well be associated with respondent factors such as age, educational attainment and self-reported health status that distinguish the Spanish from the German and English surveys. Despite these qualifications, it does appear that some VAS value differences reported in this study are of an order of magnitude that merits provisional MID status.

There remain several important issues for which there is variable evidence in the published literature. In a study of health state preferences in influenza [16], a number of complex vignettes were associated with hypothetical patients of different ages. TTO and willingness-to-pay (WTP) methods were used to elicit values for these vignettes in an internet survey (*n* = 1012). The age of the hypothetical patient varied (1, 8, 35 and 85 years of age). Evidence of preferential valuation in children was equivocal at best, and given the specific nature of the health states that were studied, it is difficult to know how far these findings can be generalised. The authors noted that “few studies have explicitly measured whether preferences for health vary by the age of the affected individual”. A separate, methodological study designed to derive distributional weights for QALYs used a DCE protocol to examine the effect of the age of the beneficiary [6]. The authors suggest that there is some evidence indicating a preference for giving “more weight to those who die at 10, 70 or 80” which they attribute to society’s desire to help the old and the young. They conclude that their results provide little evidence as to how the characteristics of recipients should be weighted when computing QALYs. Attempts to improve the robustness of utility weights appropriate for use in QALY calculations have also been reported. One study used both TTO and SG to elicit weights for 29 health states commonly encountered in children [17] from a sample of more than 4000 US adults. The combination of traditional elicitation methods and sample size suggests that these results might well satisfy utility-hungry health economists. Other generic measures developed for use with children and young people have also investigated the valuation of health. Children aged 11–17 took part in online discrete choice experiments (DCE) to derive values for the Child Health Utility 9D [5]. As well as providing evidence of the relative value that young people themselves ascribe to health states, the study also provides preliminary evidence of “systematic” differences between the values of adolescents and the adult population.

As noted in the introduction to this present study, there are many interwoven strands to any investigation of the value of health in children. There is uncertainty about the concept of health and whether this is in fact stable across all life stages from childhood into older age. It might also be that there are specific issues of valuing any child-centred attribute simply because children, as beneficiaries, have an inherently different intrinsic value? In the present study, we can only speculate about such issues given the absence of suitable empirical evidence. Without appropriate qualitative information, it is mere speculation to suggest that participants in valuation studies behave in specific ways, for example, being influenced by perceptual framing effects or the addition of personal constructs or other unobserved variables. It is without doubt a rich research field.

The present study had its origins in consideration of strategies for valuing EQ-5D-Y health states. An initial point of departure considered a stop-gap solution based on the use of adult EQ-5D-3L values. That remedy can be readily disposed of if the intended use of EQ-5D-Y is as an index measure of health status at a fixed point in time. There are large differences between values elicited when respondents are asked to think of themselves being described by a given EQ-5D-Y health state and when they are asked to imagine a child being in that same state. A high proportion of these differences are statistically significant, a result that suggests that the use of (adult) EQ-5D values in scoring child-reported EQ-5D-Y health states cannot be empirically justified. Systematic bias in the adult values will lead to the erroneous measurement of child (ill) health status. A weighted index form of EQ-5D-Y used in economic evaluation will typically be used to measure changes in health status across time, and it might be hoped that any systematic measurement bias would “wash-out” yielding results that show little difference if SELF or ADULT values were applied instead of CHILD values, but this can only be mere speculation at this point.

The study was designed to inform a judgement about whether or not values associated with EQ-5D-Y health states remained the same when those states were attached to a child rather than an adult. Given the limited number of states involved and the sampling methodologies adopted, this study essentially constitutes no more than a test of that basic issue. Despite the obvious practical difficulties encountered in this study in terms of data collection, there is strong evidence that the use of adult values to score EQ-5D-Y is contraindicated and that the proper course of action is to establish a set of weights specific to child-based EQ-5D-Y health states. This conclusion closes the door on one short-term remedy, leaving us the more substantial task of designing a valuation protocol suitable for capturing social preference weights for EQ-5D-Y health states.

## Footnotes

1 German and Spanish language versions of the questionnaire are available on request.
